# Diminished Cone Sensitivity in *cpfl3* Mice Is Caused by Defective Transducin Signaling

**DOI:** 10.1167/iovs.61.4.26

**Published:** 2020-04-21

**Authors:** Natalie S. Chen, Norianne T. Ingram, Rikard Frederiksen, Alapakkam P. Sampath, Jeannie Chen, Gordon L. Fain

**Affiliations:** 1 Zilkha Neurogenetic Institute, Department of Physiology and Neuroscience, Keck School of Medicine, University of Southern California, Los Angeles, California, United States; 2 Department of Ophthalmology, Stein Eye Institute, David Geffen School of Medicine, Los Angeles, California, United States; 3 Department of Integrative Biology and Physiology, University of California, Los Angeles, California, United States

**Keywords:** cones, transduction, mutation

## Abstract

**Purpose:**

*Cone photoreceptor function loss 3* (*Gnat2^cpfl3/cpfl3^* or *cpfl3*) is a mouse model commonly used as a functional cones null from a naturally occurring mutation in the α-subunit of cone transducin (*Gnat2*). We nevertheless detected robust cone-mediated light responses from *cpfl3* animals, which we now explore.

**Methods:**

Recordings were made from whole retina and from identified cones with whole-cell patch clamp in retinal slices. Relative levels of GNAT2 protein and numbers of cones in isolated retinas were compared between *cpfl3*, rod transducin knockout (*Gnat1^−^^/^^−^*), *cpfl3*/*Gnat1^−^^/^^−^* double mutants, and control C57Bl/6J age-matched mice at 4, 9, and 14 weeks of age.

**Results:**

Cones from *cpfl3* and *cpfl3*/*Gnat1^−^^/^^−^* mice 2 to 3 months of age displayed normal dark currents but greatly reduced sensitivity and amplification constants. Responses decayed more slowly than in control (C57Bl/6J) mice, indicating an altered mechanism of inactivation. At dim light intensities rod responses could be recorded from *cpfl3* cones, indicating intact rod/cone gap junctions. The *cpfl3* and *cpfl3*/*Gnat1^−^^/^^−^* mice express two-fold less GNAT2 protein compared with C57 at 4 weeks, and a four-fold decrease by 14 weeks. This is accompanied by a small decrease in the number of cones.

**Conclusions:**

*Cplf3* cones can respond to light with currents of normal amplitude and cannot be assumed to be a *Gnat2* null. The decreased sensitivity and amplification rate of cones is not explained by a reduction in GNAT2 protein level, but instead by abnormal interactions of the mutant transducin with rhodopsin and the effector molecule, cGMP phosphodiesterase.

Cones are the photoreceptors in the retina that are responsible for daylight vision and, in humans, for high visual acuity and color vision. The heterotrimeric G-protein, cone transducin, plays an essential role in the phototransduction pathway.^1^ The *cpfl3* mouse strain harbors a naturally occurring single-site mutation in the *Gnat2* gene, which encodes for the cone transducin α-subunit.^2^ This mutation is found in the highly conserved switch II region of the G-protein, which mediates interaction of the β- and γ-subunits with the α-subunit,^3^^,^^4^ as well as with the γ-subunit of phosphodiesterase (PDE).^4^^,^^5^ In humans, mutations in *Gnat2* are associated with achromatopsia.^6^^,^^7^ Affected individuals present with symptoms that include pendular nystagmus, severe photophobia, impaired visual acuity, and impaired color vision.^8^^,^^9^

The *Gnat2^cpfl3/cpfl3^* (*cpfl3*) strain is widely used as a model for loss of function in cone cells (for example, Allen et al.^10^), and has been used in preference to other mouse strains with mutations that cause achromatopsia (for example, Alexander et al.^11^) because cones in *cpfl3* mice have a more intact cone structure, which facilitates gene-therapy experiments. The *cpfl3* strain is advertised by The Jackson Laboratory as having “poor cone-mediated responses evident by 3 weeks of age,” and are said to “completely lack cone-mediated responses from 9 weeks of age on.” We were therefore surprised to discover robust cone responses in *cpfl3* animals in preliminary recordings from animals 2 to 4 months old.

To investigate the origin of these responses in more detail, we crossed *cpfl3* mice with *Gnat1* knockout mice, which do not express rod transducin, and thus lack a rod response. With whole-retina electroretinogram recordings, and then with patch-clamp recordings from identified cones, we found that *cpfl3* cones have a normal dark current and can respond to light, albeit with greatly reduced sensitivity. We investigated whether the lower light sensitivity is caused by (1) a decreased number of cones, or (2) a decreased amount of the GNAT2 protein. By labeling individual cone photoreceptors in the *cpfl3* strain and in normal control mice, we found an overall small decrease in the number of cones after 14 weeks. We quantified the level of GNAT2 in age-matched control C57, *cpfl3,* and *cpfl3*/*Gnat1^−^^/^^−^* mice with western blots and found that the levels of GNAT2 protein decrease by a factor of 2 to 4 as *cpfl3* mice age. Neither the modest decrease in cell number nor the decrease in the amount of GNAT2 can account for the large reduction of sensitivity in *cpfl3* cones. Our results suggest that the decreased sensitivity and altered response waveform are instead the result of a lower efficiency of transducin activation and/or PDE activation, and a slower deactivation of transducin and PDE. These effects are likely to be caused by the *cpfl3* mutation, which structural studies predict to perturb association of the G-protein α-subunit with the β- and γ-subunits, as well as with PDE6γ binding.

## Methods

### Mouse Strains

Experiments were performed in accordance with rules and regulations of the National Institutes of Health guidelines for research animals, as approved by the Institutional Animal Care and Use Committee of the University of Southern California and the University of California, Los Angeles. All animals were treated in accordance with the ARVO Statement for the Use of Animals in Ophthalmic and Vision Research. Mice were kept under cyclic light (12-on/12-off) with adlib food and water in approved cages. Male and female mice were used in approximately equal numbers and were between 1 and 4 months of age. Control mice were C57Bl/6J mice (The Jackson Laboratory, Bar Harbor, ME, USA). *Gnat1^−^^/^^−^* mice lacking the rod-specific α-subunit of the G-protein transducin were originally made in the laboratory of Janice Lem at Tufts University.^12^ These animals lacked functional rod transducin and had no detectable rod responses. *Gnat2^cpfl3/cpfl3^* (*cpfl3)* mice were obtained from Bo Chang at The Jackson Laboratory already bred into a C57BL/6J background. These mice were further mated with *Gnat1^−^^/^^−^* mice, which were also C57BL/6J. Thus, none of the mice used in this study were albino, as in the first report of this mutation.^2^ We recorded from single cones from both *cpfl3* and *cplf3*;*Gnat1^−^^/^^−^* animals, and we observed no difference in cone sensitivity or response amplitude, apart from a small rod input in *cpfl3* cones (described later).

### Genotype Verification

To test the presence of the *cpfl3* mutation, the following primers were used: *Gnat2* F (5’-CAT CGA GAC CAA GTT TCC TG-3’) and *Gnat2* R (5’-ACC ATG TCG TAG GCA CTG AG-3’). The PCR protocol was as follows: denaturation at 95°C for 3 minutes, followed by 30 amplification cycles of 30 seconds at 94°C, 1 minute at 51°C, and 1 minute at 72°C, finalized with a 7-minute extension at 72°C. PCR products were then incubated for 2 hours at 42°C with Mse1 restriction enzyme. For testing the presence of wild-type Gnat1, the following primers were used: *Gnat1* F (5’-GCC ATC TAC GGC AAC ACT CTG C-3’) and *Gnat1* R (5’-GCC GGC GGA GTC ATT GAG CTG GTA-3’). For testing the knockout of *Gnat1*, the following primers were used: TrKO F (5’-TGG ATT GCA CGC AGG TCC G-3’) and TrKO R (5’-CGG CAG GAC CAA GGT GAG ATG A-3’). PCR protocol was as follows: denaturation at 95°C for 3 minutes and 30 seconds, followed by 35 amplification cycles of 1 minute at 94°C, 1 minute at 65°C, and 1 minute at 72°C, finalized with a 7-minute extension at 72°C.

### Electrical Recording of Cone Responses

Recordings of isolated photoreceptor responses were made from whole retina with methods previously described.^13^ In brief, the retina was isolated from the eyecup, and the retinal pigment epithelium (RPE) was removed with fine tweezers. The retina was mounted in complete darkness on filter paper in a perfusion chamber, with the photoreceptor side up.^14^ One Ag/AgCl pellet electrode was placed in contact with electrode solution on the ganglion-cell side of the retina, and another electrode was placed in the solution bathing the photoreceptors. The electrodes were connected to a differential amplifier (DP-311; Warner Instruments, Hamden, CT, USA). During recording, the photoreceptors were continuously perfused with Ames’ medium (Sigma Chemical, St. Louis, MO, USA), containing an additional 1.9 g/L NaHCO_3_ and equilibrated with 95% O_2_/5% CO_2_. This solution was supplemented with 2 mM aspartic acid, 40 µM DL-2-amino-4-phosphonobutyric acid (AP4; Tocris Bioscience, Bristol, UK), and 4 mM L-lactate. The osmolarity of the medium was adjusted to 284 mOsm with a vapor-pressure osmometer (Wescore, Logan, UT, USA). Temperature was maintained at 36°C–38°C with an automatic temperature controller (Warner Instruments). Illumination was delivered with an OptoLED optical system (Cairn Research, Faversham, Kent, UK) coupled to an inverted microscope.^13^

Physiological recordings were also made from single, unlabeled cones with whole-cell patch clamp in retinal slices, as previously described.^15^ In brief, mice were euthanized by cervical dislocation after overnight dark adaptation. The anterior portion of the eye including the lens was removed, and the remaining eyecup was stored at 32°C in a custom, light-tight storage container that allowed for the gassing of solutions. For each slice preparation, half the eyecup was isolated with a #10 scalpel, and the retina was gently separated from the RPE with fine tweezers. The isolated retinal piece was embedded in 3% of low-temperature-gelling agar in Ames’-HEPES. In cold Ames’-HEPES, 200-μm thick slices were cut with a vibratome (Leica VT-1000S; Leica Biosystems, Wetzlar, Germany); the retina was cut vertically to maintain neural circuitry. Cut slices were either transferred to dishes for immediate recording or stored in the light-tight container with the remaining pieces of the eyecups. During recordings, slices were stabilized with handmade anchors, and the bath solution was maintained at 35°C ± 1°C. Cones were identified by the position and appearance of their somata, as well as from measurements of membrane capacitance and sensitivity to a moderate intensity flash. Monochromatic light was provided by ultrabright LEDs driven with a linear feedback driver (Opto-LED; Cairn Research). All light stimuli were brief (5 ms) flashes of 405-nm light, which is near the isosbestic point for mouse S and M pigments.^16^ Membrane potential was held at –50 mV, and cells were stimulated with a series of 405 nm, 5-ms flashes of increasing intensity. Intensities were converted to pigment molecules bleached (P*) from the collecting area of the cones in the slices (0.013 µm^2^, see Ingram et al.^15^). Slices were superfused at 2 mL/min in the recording chamber with Ames’ medium, which was continuously bubbled with 95% O_2_/5% CO_2_ and buffered with 1.9 g/L sodium bicarbonate to maintain pH between 7.3 and 7.4. The internal (pipette) solution was a potassium aspartate (K-Asp) solution consisting of (in mM): 125 K-Asp, 10 KCl, 10 HEPES, 5 N-methyl-D-glucamine (NMDG)-HEDTA, 0.5 CaCl_2_, 0.5 MgCl_2_, 0.1 ATP-Mg, 0.5 GTP-TRIS, and 2.5 NADPH (pH 7.3 ± 0.02 with NMDG-OH; 280 ± 1 mOsm). All values of cone membrane potential have been corrected for the liquid junction potential,^17^ which was measured to be approximately 10 mV for this internal solution.

### Western Blot Quantification

Each sample contained one extracted retina, which was homogenized and sonicated in homogenization buffer (80 mM Tris HCl pH 8.0, 4 mM MgCl_2,_ 0.5% dodecyl maltoside in H_2_O) with protease inhibitors added (0.5 mM phenylmethylsulfonyl fluoride, 0.01% P8340). Samples were incubated 10 minutes on ice. Samples were then spun down at 25,000 *g* for 10 minutes at 4°C. An equal volume of sodium dodecyl sulfate (SDS)-PAGE sample buffer was added (0.125M Tris HCl pH 6.8, 20% v/v glycerol, 4% SDS, 0.02% bromophenol blue, 0.2 M dithiothreitol in H_2_O), after which samples were boiled at 95°C for 5 minutes. Samples were loaded onto a 4% to 12% Bis-Tris gel (Thermo Fisher Scientific, Rockford, IL, USA) and run in 4% MOPS-SDS PAGE running buffer at 100 V for 2 hours. The gel was transferred to a nitrocellulose membrane overnight at 60 mA. The membrane was rinsed three times in Tris-buffered saline with Tween-20 (TBST), blocked for 1 hour in 5% bovine serum albumin, then stained overnight with antibodies for rabbit GNAT2 (1:5000, sc-105383, Santa Cruz Biotechnology, Dallas, TX, USA), rabbit PDE6C (1:2000, a gift from Nikolai Artemyev),^18^ and rabbit ARR3 1:5000, Lumi-J (a gift from Cheryl Craft).^19^ Membranes were rinsed three times in TBST and incubated for 1 hour anti-rabbit 800 nm secondary antibody (1:10,000; LI-COR, Lincoln, NE, USA). The resulting blot was imaged with the Odyssey CLx system (LI-COR). Relative levels of GNAT2 protein were compared between strains with ImageJ software (National Institutes of Health, Bethesda, MD, USA).

### Whole-Mount Immunocytochemistry

Eyes were isolated and fixed for 1 hour with 4% formaldehyde in phosphate-buffered saline (PBS). The cornea and lens were removed, and the remaining eyecup was further fixed for 2 hours in 4% formaldehyde in PBS, after which the RPE was peeled away from the retina. The isolated retinas were fixed for 1 hour in 4% formaldehyde in PBS, then cut to lay flat on a piece of nitrocellulose membrane. The retinas were washed three times in PBS and blocked for 1 hour in block solution (5% donkey serum, 0.3% Triton X-100 in PBS), then stained overnight at 4°C with either rabbit GNAT2 antibody (1:100) or rabbit cone arrestin ARR3 antibody (1:1000). The whole-mounts were rinsed once with block solution and twice with PBS, then incubated with a Alexa Fluor 488-labeled secondary antibody (1:400; Jackson Immuno Research Laboratories, West Grove, PA, USA), as well as peanut agglutinin (PNA)-rhodamine (1:300, RL-1072, Vector Laboratories, Burlingame, CA, USA). The whole-mounts were rinsed three times in PBS, then fixed for 15 minutes with 4% formaldehyde. The retinas were mounted onto a glass slide with mounting medium (Vectashield; Vector Laboratories) and imaged at 20x with a Zeiss LSM800 confocal microscope (Carl Zeiss Meditec, Jena, Germany). Images were analyzed with ImageJ and subjected to *t*-tests and ANOVA.

### Retinal Slice Immunocytochemistry

Mice were dark-adapted overnight. One group remained dark-adapted, whereas others were light-exposed. Pupils of these mice were dilated with 0.5% tropicamide and 2.5% phenylephrine hydrochloride (Akorn, Lake Forest, IL, USA) prior to light exposure (5000 lux diffuse white light for 30 minutes). Eyes were prepared immediately or after a period of dark adaptation for 2 hours. Eyes were fixed for 30 minutes with 4% formaldehyde, then washed three times in PBS, after which the cornea and lens were removed. The remaining eyecup was put into 30% sucrose for 1 to 3 hours, or until the eyecup sunk. Finally, the eyecups were embedded in OCT-compound embedding medium (Tissue-Tek; Sakura Finetek, Torrance, CA, USA) and frozen with liquid nitrogen. Slides were prepared by sectioning across the retina at 10-µM thickness with a cryostat slicing machine (Leica, Nussloch, Germany) at –20°C.

Slices were blocked for 1 hour in block solution (5% donkey serum, 0.3% Triton X-100 in PBS), and then stained overnight at 4°C with mouse TF15 antibody that recognizes both GNAT1 and GNAT2 (1:200, CytoSignal, Irvine, CA, USA) and rabbit C10C10 antibody against ARR1 (1:150). Slices were washed three times with block solution, then incubated for 1 hour with anti-mouse AlexaFluor 488- and anti-rabbit AlexaFluor 594-labeled secondary antibodies (1:200, Jackson Immuno Research Laboratories). The slices were washed three times with block solution and twice with PBS. Finally, the slices were fixed for 15 minutes with 4% formaldehyde. Slides were created with mounting medium containing DAPI (Vectashield, Vector Laboratories, Burlingame, CA, USA) and imaged at 20x with a Zeiss LSM800 confocal microscope (Carl Zeiss Meditec).

## Results

**Figure 1.**
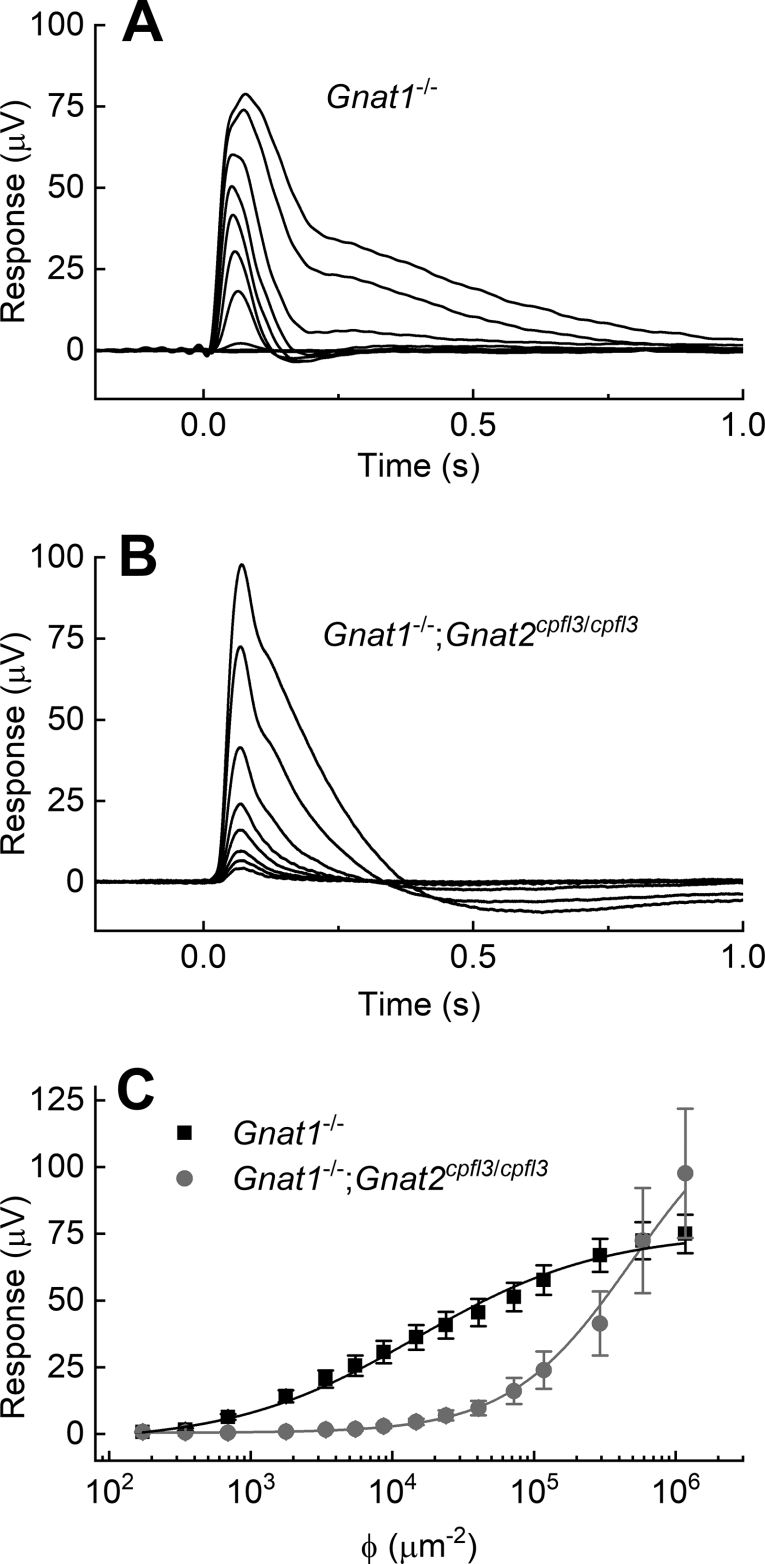
Isolated whole-retina photoreceptor recordings from dark-adapted retinae of *Gnat1^−^^/^*^−^ and *Gnat1^−^^/^^−^;cpfl3* mice. (**A**) *Gnat1^−^^/-^* responses to flash stimuli (505 nm, 10 ms) of increasing intensity. The flash intensities used were 170, 350, 690, 3400, 8700, 24,000, 72,000, 120,000, 59,000, and 1,200,000 photons (ϕ) µm^−2^. The response family is an average from 10 retinae. (**B**) *Gnat1^−^^/^^−^;cpfl3* responses to flash stimuli (505 nm, 10 ms) of increasing intensity. The flash intensities used were 15,000, 24,000, 41,000, 72,000, 12,000, 29,000, 57,000, and 1,200,000 ϕ µm^−2^. The response family is an average from four retinae. (**C**) Response plotted as a function of stimulus intensity from *Gnat1^−^^/-^*retinae (*black filled squares*, *n* = 10) and *Gnat1^−^^/^^−^;cpfl3* retinae (*red filled circles*, *n* = 4). The data were fitted with Hill equations (Equation 1) with the best fitting parameters of: *Gnat1^−^^/^^−^*, *I_½_* = 15,000 and *n* = 0.67; and *Gnat1^−^^/^^−^;cpfl3*, *I_½_*= 490,000 and *n* = 1.01. *Error bars* are SEM.

In experiments attempting to study a pure rod response from mouse retina in the absence of cone activity, we recorded from the retinas of *cpfl3* mice 2 to 3 months of age because these animals were reported to have virtually no photopic electroretinogram as soon as 1 month after birth (see Fig. 3B of Chang et al.^2^). We began by recording the isolated massed photoreceptor response from the whole retina of *cpfl3* mice (see Methods section), expecting to see only rod responses. We were surprised, however, to detect an apparent rapidly decaying cone contribution to the response preceding the more slowly decaying rod component (recordings not shown). To isolate this cone component and compare it to normal cone responses, we then recorded from the retinas of mice that were *Gnat1^−^^/^^−^* and lacked rod function^12^ or were both *cpfl3* and *Gnat1^−^^/^^−^*, which harbored the *cpfl3* mutation but again lacked rod function. The *Gnat1^−^^/^^−^*;*cpfl3* retinas showed robust cone responses (Fig. 1B), nearly as large as those from *Gnat1^−^^/^^−^* retinas.^13^ The waveforms of the responses exhibited some dissimilarities, but no effort was made to explore this phenomenon in detail.

There was, however, an important difference between the cone responses in *Gnat1^−^^/^^−^* and *Gnat1^−^^/^^−^*;*cpfl3* retinas. The *Gnat1^−^^/^^−^*;*cpfl3* responses were much less sensitive to light (Fig. 1C). The peak amplitude of the responses from Figs. 1A through 1B were fitted with the Hill equation:

(1) $$\begin{array}{c} \frac{r}{r_{max}}=\frac{I^{n}}{\left(I^{n}+I_{1/2}^{n}\right)} \end{array}$$

where, *r* is the peak amplitude of the response to a given stimulus, *r_max_* is the maximum value of *r* at the brightest stimulus, *I* is the number of photons per µm^2^, and *I_½_* is the value of *I* required to produce a half-maximal response. The best-fitting values of *n* and *I_½_* are given in the legend to Figure 1, and predict half-maximal responses (*r*/*r_max_* = 0.5) of approximately 1.6 × 10^4^ photons for *Gnat1^−^^/^^−^* and 4.9 × 10^5^ photons for *Gnat1^−^^/^^−^*;*cpfl3*, a difference of about 30-fold. These values are approximate because saturation may not have been reached (*r* = *r_max_*) in the *cpfl3* retinae.

**Figure 2.**
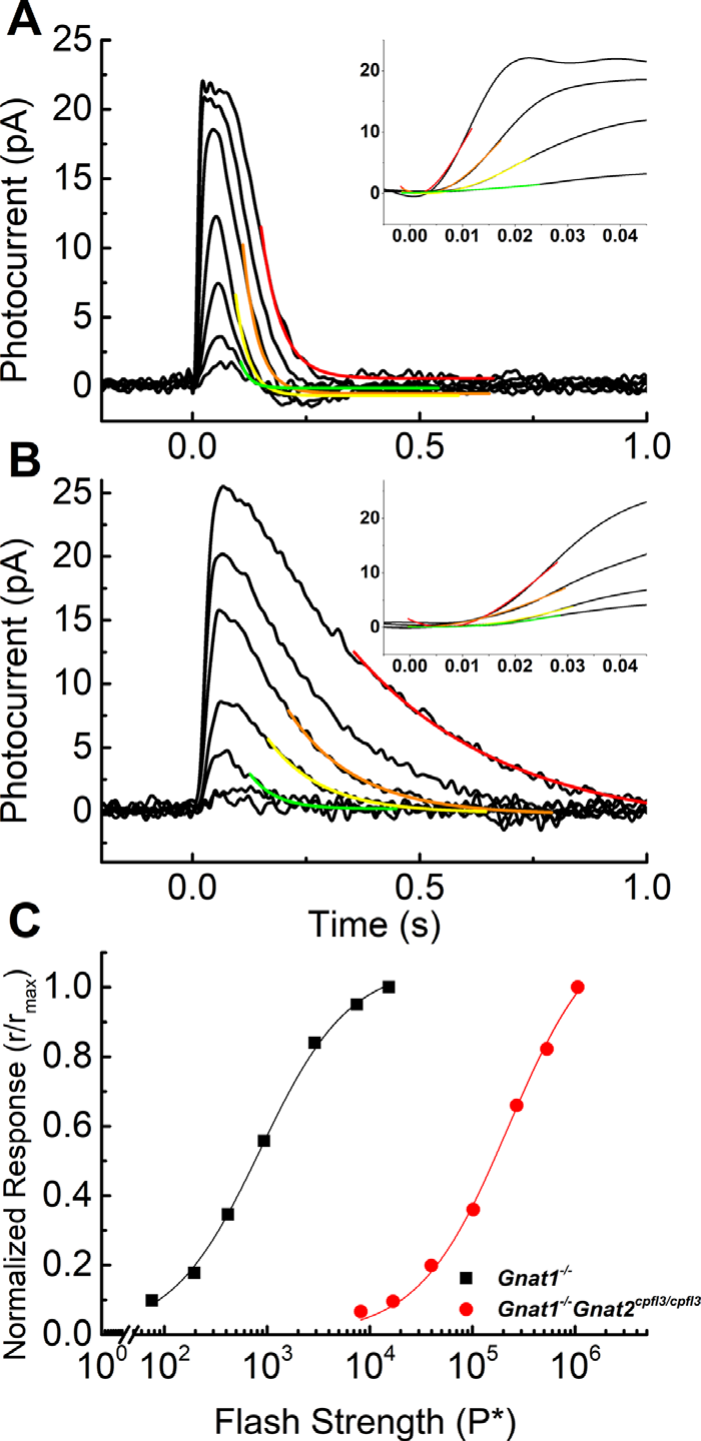
Responses of single cones from *Gnat1^−^^/^^−^* and *Gnat1^−^^/^^−^;cpfl3* retinas. Cones were identified from visual clues in the dark-adapted retina^15^ and voltage clamped at –50 mV. Light stimuli were 5 ms in duration provided by an LED at 405 nm, near the isosbestic point of mouse S and M cones. (**A**) Mean response of 10 cones from *Gnat1^−^^/^^−^* mice. Stimuli were 76, 190, 420, 940, 2900, 7500, 1.5 × 10^4^ pigment molecules bleached (P*) per flash. Colored lines are fits to single-exponential decay functions, with the following time constants (in ms) and R^2^ values (from smallest response to largest): 44.1, 0.98; 33.0, 0.94; 28.7, 0.89; and 20.5, 0.60. (**B**) Mean response of five cones from *Gnat1^−^^/^^−^;cpfl3* mice. Stimuli were 8200, 1.7 × 10^4^, 4.0 × 10^4^, 1.0 × 10^5^, 2.7 × 10^5^, 5.3 × 10^5^, 1.1 × 10^6^ P* per flash. Colored lines are fits to single-exponential decay functions, with the following time constants (in ms) and R^2^ values (from smallest response to largest): 333, 0.99; 153, 0.99; 150, 0.99; 57, 0.76. *Insets to* ***A****and* ***B***: The rising phases of responses are shown to the same four stimuli used to fit exponential decay, with the same color coding. Responses were fit with Equation (3) to derive *A*, the amplification constant (*colored lines*). (**C**) Peak current responses normalized to the maximum peak response and plotted against P*. Data were fit with Equation (2) with best-fitting values of *I_½_* of 860 P* for *Gnat1^−^^/-^* (*black*) and 2.2 × 10^5^ P* for *Gnat1^−^^/^^−^;cpfl3* (*red*).

To explore the physiology of this response in more detail, we made patch-clamp recordings from identified cones in retinal slices.^15^ As we and others have previously shown, cones in control C57Bl/6J retinae receive an input of variable amplitude from rods presumably through rod-cone gap junctions.^20^ In preliminary experiments from *cpfl3* retinae, rod responses could be recorded from *cpfl3* cones at dim light intensities, indicating intact rod/cone gap junctions. We therefore recorded from *cpfl3* cones in *Gnat1^−^^/^^−^* retinas, which are almost entirely devoid of rod function.^12^ Recordings from a typical cone from a *Gnat1^−^^/^^−^* retina are shown in Figure 2A, in response to 5-ms flashes of a series of increasing brightness. These records show the change in current in response to light at a cone-membrane holding potential of –50 mV, which is near the resting potential of a *Gnat1^−^^/^^−^* cone.^15^ Responses of *Gnat1^−^^/^^−^* cones are quite similar to those of *Cx36^−^^/^^−^* cones lacking connexin36 gap junctions, and of a small proportion of C57Bl/6J cones lacking any rod component.^15^ The responses in Figure 1A can therefore be taken to be representative of voltage-clamp current responses of normal mouse cones.

When we then recorded from *cpfl3* cones that were also *Gnat1^−^^/^^−^*, we were surprised to observe responses of normal amplitude (Fig. 2B). Mean values of peak currents from the cones of Figures 2A through 2B were 22.3 ± 0.9 pA (*n* = 10) and 26.7 ± 4.6 pA (*n* = 5) and were not significantly different. The responses of the *Gnat1^−^^/^^−^*;*cpfl3*, cones did, however, differ from those of normal cones in several other ways. In the first place, the *cpfl3* cones were a factor of over 100-fold less sensitive. In Figure 2C, we compare response-intensity curves of *Gnat1^−^^/^^−^* cones with *Gnat1^−^^/^^−^*;*cpfl3* cones. The data in both cases have been fitted with a Michaelis-Menten equation, which is Equation (1) with *n* = 1:

(2) $$\frac{r}{r_{max}}=\frac{I}{\left(I+I_{1/2}\right)}$$

*I_½_* is now the value of P* required to produce a half-maximal response and was used as a measure of sensitivity. Our best-fitting values were approximately 860 P* for normal cones and 2.2 × 10^5^ P* for *cpfl3* cones, both on a *Gnat1^−^^/^^−^* background. By this measure, the *cpfl3* cones were approximately a factor of 250 less sensitive than normal cones, even though the value of the circulating (dark) current was approximately the same. This difference in sensitivity is even larger than the difference we detected in Figure 1C from whole-retina recordings, perhaps the result of a difference in recording conditions. Whole-retina recordings reflect changes in cone voltage, whereas the recordings in Figure 2 are recordings of voltage-clamp current and give changes in cone outer-segment conductance.

The second difference was the rate of rise of the light response. To quantify this difference, we estimated the amplification constants of the cones in the following way.^21^ The first third to half of the rising-phase of each flash response before digital filtering was fit with:

(3) $$r=r_{max}\left(1-e^{\left(-a{\left(t-t_{eff}\right)}^{2}\right)}\right)$$

where *t_eff_* (in seconds) is a time delay from stimulus onset to initiation of the photoresponse (see insets to Figs. 2A, 2B). To calculate the amplification constant (*A*) for a specific flash response, the derived parameter *a* from Equation (3) was multiplied by 2 and divided by the number of pigment molecules (P*) activated by the flash. With this method, we have previously estimated *A* for *Gnat1^−^^/^^−^* cones to be about 2 s^-1^.^15^ A similar analysis for *cpfl3*;*Gnat1^−^^/^^−^* cones gave a mean value of 0.02 s^-1^. The ratio of these values is approximately 100, indicating a profound decrease in the rate of activation of the cone transduction cascade in the *cpfl3* cones, either at the level of transducin activation by R* or PDE6 activation by transducin, or both.

A final difference is the slower rate of decay of the *cpfl3* cones. The decay phase of responses to dim and moderate-intensity stimuli (giving responses less than half of maximal) were fitted to a single-exponential decay function to derive the time constant of recovery, τ_REC_. Fits to mean responses are shown in Figures 2A through 2B, with values of time constants and R^2^ values given in the figure legend. We have previously shown that for *Gnat1^−^^/^^−^* cones, the value of τ_REC_ is between 30 and 40 ms. For the sample of cones used for Figure 2A, the global mean calculated from all of cones and all of the responses was 31 ± 2 ms. For the *cpfl3*;*Gnat1^−^^/^^−^* cones of Figure 2B, the global mean for τ_REC_ was 160 ± 19 ms, five-fold slower. For both the *Gnat1^−^^/^^−^* cones and *cpfl3*;*Gnat1^−^^/^^−^* cones, there was a tendency for the time constant to become progressively shorter as the flash was made brighter (see legend to Fig. 2), a trend we did not explore in detail.

Model calculations (Reingruber, Ingram, Griffis & Fain, submitted for publication) indicate that the slowing of decay produced by the *cpfl3* mutation cannot be produced by a change in the rate of activation of transducin by R*. The number of transducins produced within the lifetime of R* would decrease, but these transducins would decay at the same rate as transducins in C57Bl/6J cones. If the *cpfl3* mutation were to decrease the rate of activation of PDE by T*, the rate of response decay could be affected, but much larger changes would also occur in the time-to-peak amplitude and other aspects of the waveform of the response than those we show in Figure 2B. For this reason, we believe that the most likely explanation of the slowing of decay is an alteration of the interaction between the mutant transducin α-subunit and the γ-subunit of the PDE, a notion supported by x-ray crystallographic studies that show the close proximity of the *cpfl3* mutation (D200N) to nearby γ-PDE binding residues.^5^

### The Number of Cones is Similar in *cpfl3* and C57 Control Mice

**Figure 3.**
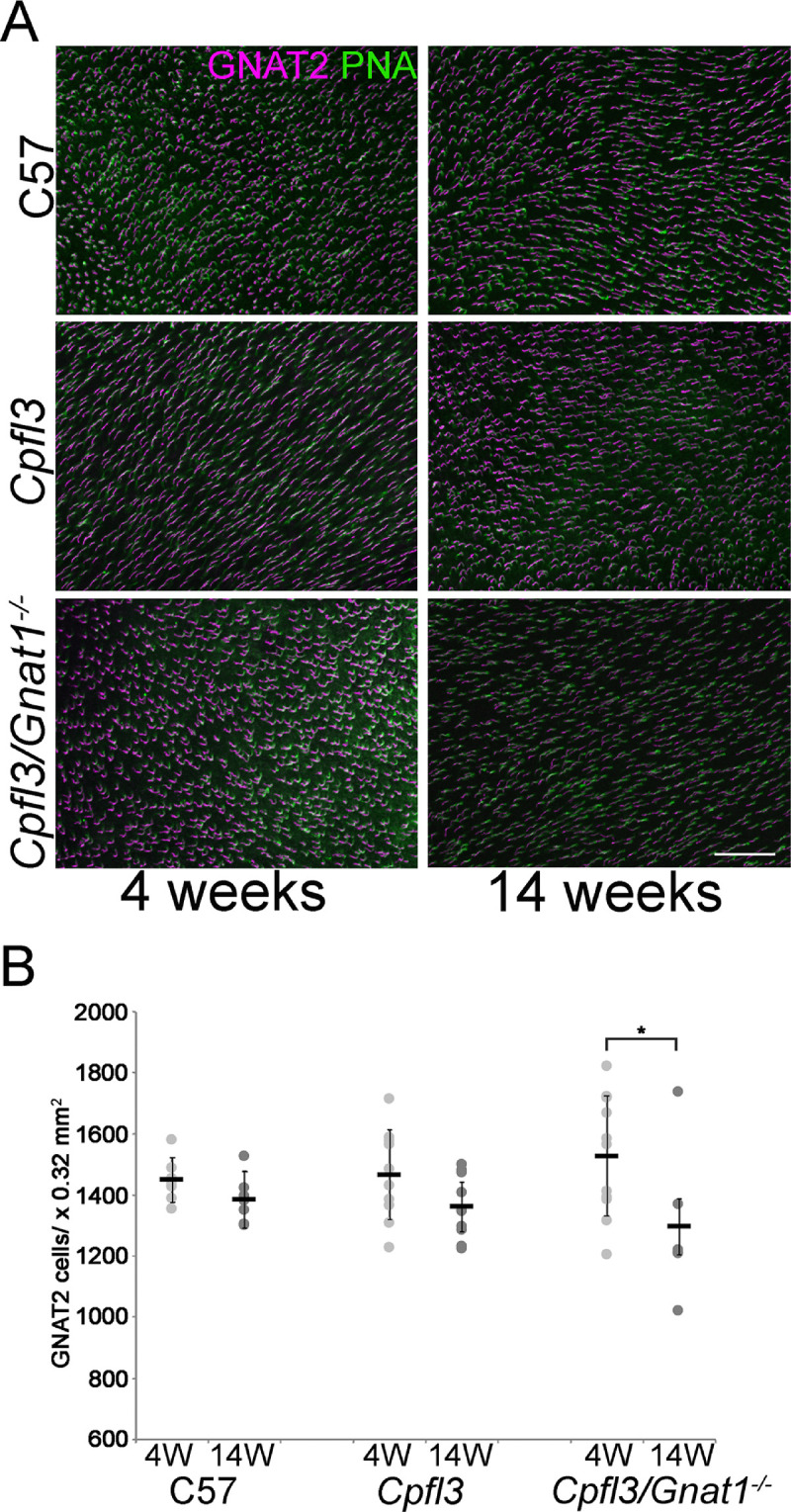
The *cpfl3* mutation has a minor effect on cone survival. (**A**) Flat-mounted retinas from 4-week and 14-week-old mice were stained with markers for cone transducin α-subunit (GNAT2, magenta) and peanut agglutinin (PNA, *green*). *Scale bar* = 50 µm. (**B**) The number of GNAT2-positive cones were quantified with the image processing package Fiji (ImageJ) ImageJ and the values plotted (mean ± SD, *N* = 5–9, asterisk = *P* < 0.05 by 2-tailed Student's *t*-test).

Because the amplitudes of responses from whole retina were nearly the same in normal and *cpfl3* retinas (Figs. 1A, 1B), and the responses of individual cones were nearly the same in amplitude (Figs. 2A, 2B), the number of cone photoreceptors in *cpfl3* mice would seem not to be much altered. To investigate this phenomenon in detail, retinas were isolated from *cpfl3*, *cpfl3/Gnat1^−^^/^^−^*, and control C57 mice at 4 and 14 weeks of age. Flat mounts were prepared, and the cones were labeled with two different cone-specific markers: GNAT2 and peanut agglutinin PNA.^22^ The cones were readily colabeled by both in all strains at both 4 and 14 weeks (Fig. 3). We observed an age-dependent decrease in the number of GNAT2/PNA-positive cones at 4 week- versus 14-week-old *cpfl3/Gnat1^−^^/^^−^* mice (Fig. 3B, *P* < 0.05; *n* = 9–12). Despite this small age-dependent decrease, the overall number of cones is similar between *cpfl3* mutant and control retinas.

### Western Blot Quantification: *cpfl3* Strain has Reduced Amount of GNAT2 Protein

**Figure 4.**
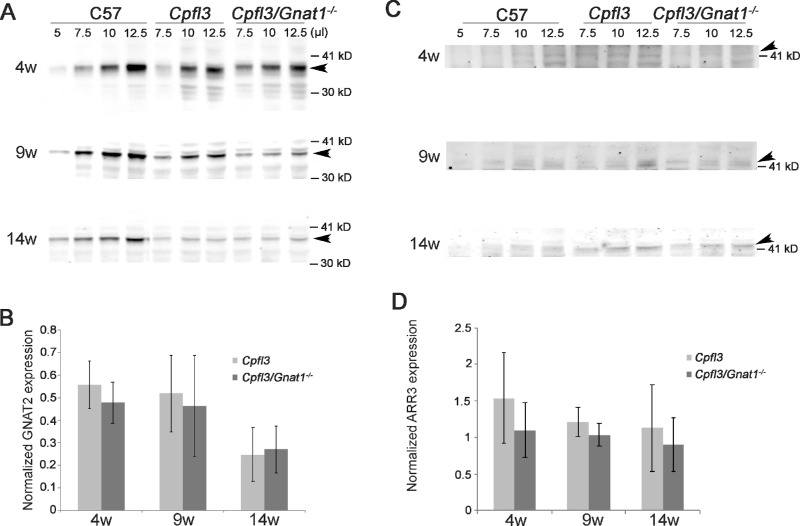
GNAT2 protein expression is lowered in *cpfl3* cones. Western blots of whole-retinal homogenates from control C57Bl/6J (C57), *cpfl3*, and *cpfl3/Gnat1* mice at 4, 9, and 14 weeks of age probed with antibody to GNAT2 (**A**) and cone arrestin (**C**, *HUGO* ARR3 *= systematic name* ARR4). Signals were quantified and normalized to that of C57 and plotted in **B** and **D**, respectively. Values shown are mean ± SD (*N* = 5–9).

To see if the decrease in cone sensitivity could be explained by a reduction in the amount of GNAT2 protein, western blots were performed to compare the relative amount of GNAT2 in retinas of *cpfl3* strains versus C57 strains. Whole-retinal extracts were prepared from *cpfl3*, rod transducin knockout (*Gnat1*), *cpfl3*/*Gnat1* double knockouts, and control C57 age-matched mice at 4, 9, and 14 weeks of age. Quantification of signal intensities showed that *cpfl3* and *cpfl3/Gnat1* mice have two-fold less GNAT2 protein compared with C57 mice at 4 and 9 weeks old. By 14 weeks old, the level of GNAT2 further decreased by approximately four-fold (Figs. 4A, 4B). To test whether other cone phototransduction proteins are affected, we examined the level of cone arrestin using western blots (*HUGO* ARR3 *= systematic name* ARR4, Figs. 4C, 4D). Quantification of arrestin signals show that *cpfl3* strains and C57 display similar expression levels.

### Localization of GNAT2 in Cones is not Altered by the *cpfl3* Mutation

**Figure 5.**
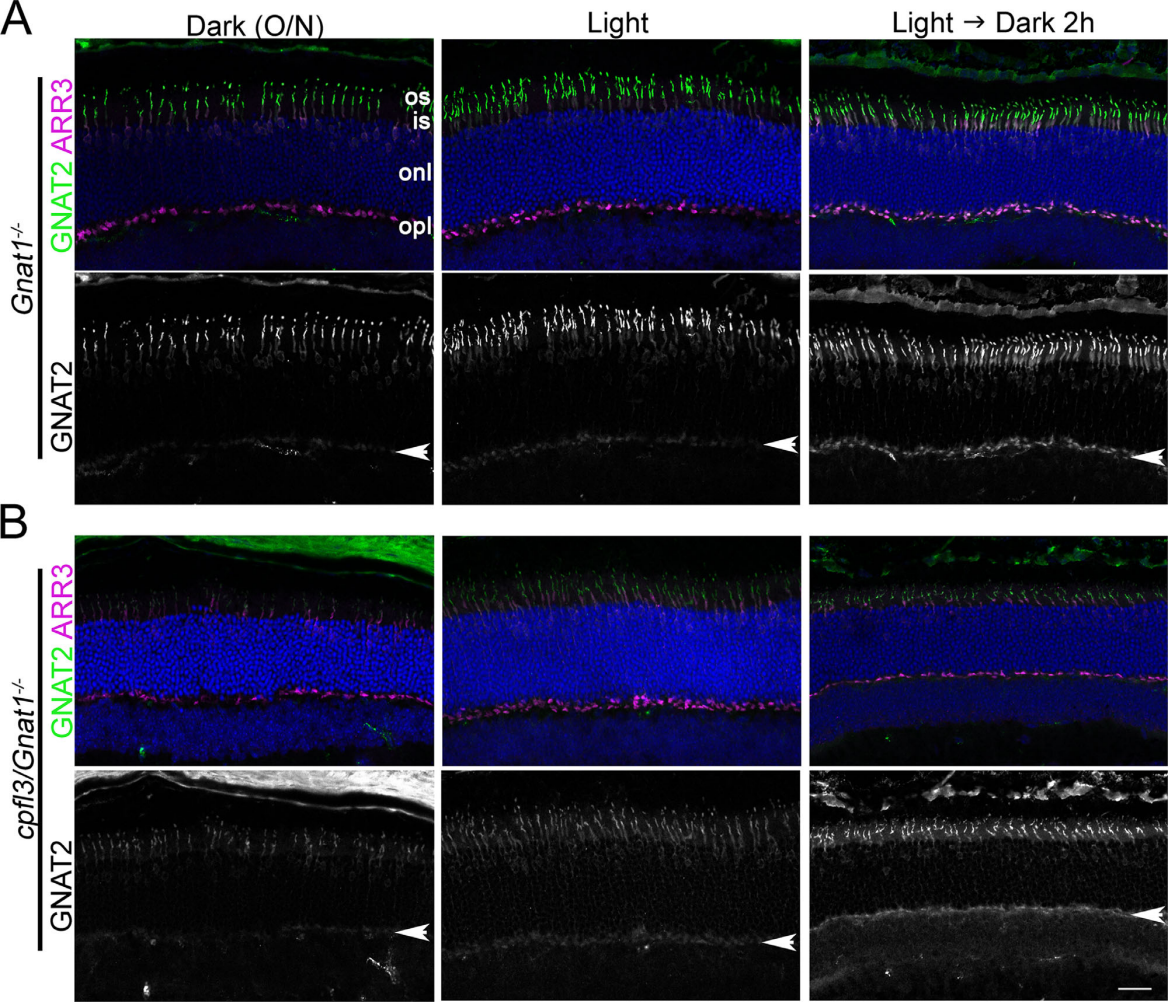
GNAT2 localization was not affected by the *cpfl3* mutation under dark- and light-exposed conditions. Retinal sections from dark-adapted or light-exposed (5000 lux, 30 minute) control *Gnat1^−^^/^^−^* (**A**) and *cpfl3/Gnat1^−^^/^^−^* mice (**B**) were stained with GNAT2 (*green*) and cone arrestin (*magenta*) antibodies. The retinal layers are outer segment (os), inner segment (is), outer nuclear layer (onl), and outer plexiform layer (opl). The position of the opl is marked by the *arrow*. *Scale bar* = 20 µm.

As can be seen in Figure 5, GNAT2 is predominantly localized to the cone outer segment in dark-adapted retinas from control *Gnat1^−^^/^^−^* (Fig. 5A) and *cpfl3/Gnat1^−^^/^^−^* cones (Fig. 5B). Light exposure caused some GNAT2 immunoreactivity to appear in the outer plexiform layer (Fig. 5, arrows). This pattern is also similar between control *Gnat1^−^^/^^−^* and *cpfl3/Gnat1^−^^/^^−^* cones. These results suggest that although the *cpfl3* mutation affected cone transducin function, it did not affect the localization pattern of GNAT2.

## Discussion

### The *cpfl3* Cones Display Robust Responses, Albeit Slower, and with Reduced Sensitivity

Our results show that cones in *cpfl3* mice 2 to 3 months of age display responses to light of nearly normal amplitude, although with slower activation, slower decay, and greatly reduced sensitivity. The *cpfl3* mouse line is not a cone-transducin knockout line but has functional cones although with aberrant physiology. We further show that the number of cones in the *cpfl3* retina is not much reduced from the number in normal retinas up to at least 4 months of age, and the amount of transducin in *cpfl3* retinas is only modestly decreased by a factor of at most 4. The relationship between the amount of transducin and photoreceptor sensitivity is unclear, with evidence indicating a simple linear relationship^23^ or a decidedly nonlinear relation such that the decrease in sensitivity is much less than the decrease in transducin concentration.^24^ Such a nonlinear relation could exist if the rate of activation were less affected by the rate of encounter of holo-transducin with light-activated rhodopsin than by the rate of production of activated transducin-α once binding has occurred. There is no indication, however, that a two-fold to four-fold decrease in transducin concentration could produce a larger than two to four times decrease in sensitivity, much less the 30 to 250 times decrease we have observed for *cpfl3* cones. We conclude that the effect of the *cpfl3* mutation on the rate of activation and sensitivity must result from some more specific alteration in the function of the transducin α-subunit.

Rod and cone transducins are heterotrimeric G-proteins encoded by distinct α-, β- and γ-subunits. Although loss of the α-subunit almost entirely abolishes the light response in rods^12^ and cones,^1^ knockouts of the β- and γ-subunits do not.^25^^,^^26^ In rods lacking the transducin γ-subunit, the α-subunit level is reduced, with an even greater reduction in the amplification efficiency.^25^ Similarly, knockout of the cone transducin β-subunit, Gβ3, led to a reduced amplification constant.^26^ Thus it has been proposed that heterotrimeric association (1) confers structural stability to the α-subunit, and (2) increases the efficiency of R*-transducin activation. Interestingly, the *cpfl3* mutation (Asp200Asn) occurs in the switch II domain. This domain is part of the highly conserved switch I, II, and III domains, which contain transducin-βγ as well as PDE6γ-interacting sites, which change conformation on GDP/GTP exchange.^27^ Together, these observations support the notion that the Asp200Asn mutation may have diminished the interaction between the α- and βγ-subunits. By doing so, it may have produced structural instability of the α-subunit, as well as a reduction in the efficiency of transducin activation by light-activated visual pigment. The Asp200 position is also in close proximity to key residues that interact with PDEγ. Thus, the Asp200Asn mutation may also reduce the efficiency of PDE activation and delay transducin deactivation, consistent with our observations.

### Mutant *cpfl3* GNAT2 Shows Correct Localization Patterns in the Cone Photoreceptor

In darkness, the rod transducin heterotrimer is predominantly localized to the membranous outer-segment compartment via the synergistic action of the lipid modifications on the α- and γ-subunits.^28^ Activation of transducin by R* causes dissociation of the heterotrimeric G-protein, weakening the synergy of the membrane attachment of the GTP-loaded α-subunit and the βγ-subunits. As a result, these subunits diffuse to the aqueous inner-segment compartments. Similarly, cone transducin exhibits this light-dependent translocation as seen here and also in a previous report^29^^,^ (but see also^30^). If association of the cone transducin heterotrimer in the GDP-bound state is required for outer-segment membrane binding, then one would expect the *cpfl3* mutation, by affecting βγ interaction, to be mislocalized to the cytoplasmic compartments in dark-adapted retinas. However, this appears to not be the case, suggesting that the *cpfl3* mutation may not have caused subunit dissociation of the heterotrimeric G-protein in its basal state.

### Studies Using *cpfl3* Mice that Assume a Functional Null Phenotype

A concern with the *cpfl3* model is its wide use in the field as a cone functional null. The 30- to 250-fold reduction in sensitivity would not necessarily have a significant effect on studies attempting to isolate the contribution of rod photoreceptors to retinal function over a limited range of light intensities,^31^^–^^33^ or near the threshold for normal cone function.^34^^,^^35^ However, residual responses in the cones may undermine attempts to evaluate the role of cones at high light intensity. For example, circadian studies using *cpfl3* mice showed different effects on entrainment at high light intensity when compared with another model of cone functional loss, *Cnga3^−^^/^^−^* mice.^36^ The ability of *cpfl3* mice to entrain at high but not low light intensities (unlike *Cnga3^−^^/^^−^* mice which could not entrain at either intensity) might be explained if *cpfl3* cones displayed residual responses that were communicated downstream to intrinsically photosensitive retinal ganglion cells. In addition, studies to evaluate residual retinal function under conditions in which all known phototransduction has been ablated would similarly be compromised. For example, Allen et al.^10^ interpreted the residual responses in *Gnat1^−^^/^^−^*;*cpfl3* mice as potentially originating in rods because of the low level of expression of Gnat2 in those cells. A more complete model of Gnat2 loss would be preferable in studies of this nature, to eliminate cone function fully.^1^

Mutations in GNAT2 have been observed in patients with achromatopsia,^8^^,^^9^ and some of these mutations map to the switch domains.^6^^,^^7^ Although the *cpfl3* strain is not completely lacking cone function, it could still be a useful model to understand how transducin mutations lead to reduced photoreceptor function.
